# Analysis of status quo and influencing factors for health-promoting lifestyle in the rural populace with high risk of cardiovascular and cerebrovascular diseases

**DOI:** 10.1186/s12872-023-03129-7

**Published:** 2023-03-08

**Authors:** Jing Li, Jia Song, Xia-Ling Zhu, Mei-Fen Chen, Xu-Fang Huang

**Affiliations:** 1 Department of Nursing Administration, Lishui Central Hospital, No. 289 Kuocang Road, Liandu District, Lishui, 323000 China; 2 Department of Respiratory Medicine, Lishui Central Hospital, Lishui, 323000 China; 3 Lishui Central Hospital, No. 289 Kuocang Road, Liandu District, Lishui, 323000 China

**Keywords:** Cardiovascular and cerebrovascular diseases in rural areas, Health-promoting lifestyle, High-risk population, Influencing factors

## Abstract

**Objective:**

To explore the status quo and influencing factors for health-promoting lifestyle in the rural populace with high risk of cardiovascular and cerebrovascular diseases, and to provide reference for developing primary prevention strategies for cardiovascular and cerebrovascular diseases.

**Method:**

Questionnaire-based survey of 585 cases of high-risk cardiovascular and cerebrovascular population in 11 administrative villages in Fuling of Lishui city was conducted using the Health Promoting Lifestyle Profile-II (HPLP II), Perceived Social Support from Family Scale (PSS-Fa), General Health Questionnaire (GHQ-12), and other questionnaire tools.

**Results:**

The total score of the health-promoting lifestyle in the rural populace with high risk of cardiovascular disease is 125.55 ± 20.50, which is at an average level, and the mean scores of each dimension in descending order are—nutrition, interpersonal support, self-actualization, stress management, health responsibility, and exercise. Monofactor analysis revealed that age, education level, marriage, monthly per capita household income, physical activity based on the International Physical Activity Questionnaire (IPAQ), family support function, carotid intima-media thickness, and blood pressure were influencing factors for the health-promoting lifestyle in the rural populace with high risk of cardiovascular and cerebrovascular diseases (*P* < 0.05). Multiple stepwise regression analysis showed that monthly per capita household income, family support function, physical activity based on the IPAQ, and education level were positively correlated with the level of the health-promoting lifestyle.

**Conclusion:**

The health-promoting lifestyle level of the rural populace with high risk of cardiovascular and cerebrovascular diseases needs to be improved. When assisting patients to improve their health-promoting lifestyle level, it is imperative to pay attention to improving patients' physical activity level, emphasizing the influence of the family environment on patients, and focusing on patients with economic difficulties and low education level.

## Introduction

Cardiovascular and cerebrovascular diseases are the leading causes of mortality in China, and have become a major public health and social problem that affect the health of the Chinese people and hinder social and economic development [1]. The ratio of deaths in rural areas from cardiovascular and cerebrovascular diseases to all causes of death is 45.5%, compared with 43.16% in cities, and the phenomenon of higher cardiovascular and cerebrovascular mortality in rural areas compared to cities has been persistent for a long time. Thus, rural areas have become a key intervention area for prevention and control of cardiovascular and cerebrovascular diseases [2, 3]. Cardiovascular and cerebrovascular diseases are behavior-related diseases, and behavior-related influencing factors include diet, low physical activity, smoking, alcohol abuse, metabolic factors, psycho-emotional factors, family support, sleep, etc. Early intervention through a health-promoting lifestyle for people with high risk of cardiovascular and cerebrovascular disease is the key to prevent and control the occurrence of cardiovascular and cerebrovascular disease, and can prevent more than 80% of occurrences [4, 5]. Therefore, the purposes of this study are to investigate and evaluate the status quo and influencing factors of health-promoting lifestyle in the rural populace with high risk of cardiovascular and cerebrovascular diseases, improve the level of health-promoting lifestyle of the rural populace with high risk of cardiovascular and cerebrovascular diseases, and provide a reference basis for primary prevention of cardiovascular and cerebrovascular diseases in rural areas.

## Participants and methods

### Study participants

From May to July 2021, 585 persons from rural areas with high risk of cardiovascular and cerebrovascular diseases from 11 administrative villages of Fuling Street affiliated to the member unit of Lishui Central Hospital medical facility were selected as study participants using the convenience sampling method. Inclusion criteria: Based on the results of the *National Major Public Health Service Project—Comprehensive Intervention Investigation Form for Community and Township Populations on Risk Factors of Cardiovascular and Cerebrovascular Diseases* that we used in this study in the initial stage, the high-risk population for cardiovascular and cerebrovascular diseases (those with ≥ 3 of the following risk factors or previous history of stroke or coronary heart disease) whose occupation was farmers were screened: (1) hypertension ≥ 140/90 mmHg or taking antihypertensive drugs; (2) dyslipidemia or unknown; (3) diabetes; (4) smoking; (5) obesity with BMI ≥ 26 kg/m^2^; (6) infrequent physical activity (physical activity criteria is ≥ 3 times per week for ≥ 30 min for more than 1 year; those engaged in moderate to heavy physical labor are considered to undergo regular physical activity); (7) family history of cardiovascular and cerebrovascular disease. The following conditions were also satisfied: (1) possess a certain level of understanding and communication ability and able to express their wishes correctly; (2) possessing a telephone contact number of their own or of their immediate family members; (3) usually residing in a rural area; (4) signed informed consent form for voluntary participation in this study. Exclusion criteria: (1) severe dysfunction of important organs such as liver, kidney, and lung; (2) hearing or speech impairment; (3) cognitive dysfunction; (4) mental disorders.

### Study tools

#### General information questionnaire

A self-designed general information questionnaire was used for patient assessment, which included general demographic information, health economic indicators, lifestyle assessment, and physiological related indicators. (1) General demographic information: gender, age, education level, residence status, marriage status, per capita monthly household income, etc.; (2) Health economic indicators: visit frequency, medical costs, duration of hospitalization, prevalence of hypertension, diabetes, stroke, and other diseases, daily management, etc.; (3) Lifestyle assessment: smoking history, alcohol consumption history, dietary habits, etc.; (4) Physiological related indicators: blood glucose, blood lipids, blood pressure, uric acid, homocysteine, blood pressure, body mass index, waist circumference, neck circumference, electrocardiogram, cervical vascular ultrasound, etc.

#### Health-promoting lifestyle profile II (HPLP II)

This profile was developed by Deniz et al. [6] to assess the frequency of people adopting health-promoting behavior, and can be used to comprehensively evaluate the health-promoting lifestyle of the study participants. It includes six dimensions—self-actualization, health responsibility, exercise, nutrition, interpersonal support, and stress management. A total of 52 items are contained in the profile with a 4-point Likert scale, with scores ranging from 1–4, with 1 indicating never, 2 indicating sometimes, 3 indicating often, and 4 indicating routinely, with the score ranging from 52–208; a higher score indicates higher level of health-promoting lifestyle. The health-promoting lifestyle is divided into 4 levels, with a score of 52–91 meaning poor, 92–131 meaning average, 132–171 meaning good, and 172–208 meaning excellent. The profile has good reliability and validity with Cronbach's α coefficient of 0.94, and the range of Cronbach's α coefficient for each dimension is 0.79–0.87.

#### Perceived Social Support from Family Scale (PSS-Fa)

This scale was developed by Procidano et al. in the United States and is currently used to assess family function and family support in China [7]. The scale contains 15 items, and each item can have a score of 1 for "yes" and 0 for "no"; some items have the scores in reverse. The scale has a maximum score of 15, with a higher score representing a better level of family support. Scores ≥ 10 indicate high family support and < 10 points indicate low family support; the Kuder-Richardson Formula 21 value for the scale is 0.75 [8].

#### General Health Questionnaire (GHQ-12)

This questionnaire is widely used for screening of community populations and mental health and has been localized by Chinese scholars. It has good reliability and validity [9]. The scale contains 12 questions, with 4 options for each question, and is scored using a double-peaked scale (0–0–1–1). It has a total score ranging from 0–12, with a higher score indicating higher possibility of psychological problems and poorer level of mental health. A cumulative score ≥ 4 for the 12 questions is considered to indicate mental health status detection [10].

#### International Physical Activity Questionnaire (IPAQ)

This questionnaire is used to measure the physical activity level of adults, and is widely used in the world. It is divided into a long questionnaire and a short questionnaire, wherein the Chinese version of the short questionnaire includes 7 questions. As the questionnaire is widely used, it has good reliability and validity [11].

#### Pittsburgh Sleep Quality Index (PSQI) [12]

This scale is suitable for a comprehensive assessment of sleep status and has been localized with internal consistency reliability of 0.84 and retest reliability of 0.81.

### Data collection and analysis

The questionnaires were distributed to the people while they were undergoing free physical examinations at the community health service centers for rural residents using the convenience sampling method with a uniform guiding expression. All enrolled participants were administered the questionnaire on the principle of fully informed consent and voluntary participation and all investigators were homogenously trained and assessed. The questionnaire survey was conducted via face-to-face questioning, filled out by the investigator in batches within a specified period, and the content of the scales were checked with the investigator after the scales were completed by the respondents; any errors or omissions were corrected or supplemented immediately. A survey team leader was set up to check the authenticity and completeness of each questionnaire day by day from the start of the official survey. The final data was input by a designated nurse from the Heart and Brain Prevention Office and checked by a full-time statistician for consistency and logical errors, and any extreme values were verified. A total of 595 questionnaires were distributed in this study, and 585 valid questionnaires were recovered, with a valid recovery rate of 98.3%.

### Statistical methods

The sample size of the multifactorial analysis design is estimated to be at least 10 times the number of variables [13, 14]. There are a total of 52 questions in the Health Promoting Lifestyle Profile-II (HPLP II); therefore, the sample size should be greater than 52*10 = 520 cases. A database was established using Epidata 3.1, data input was performed by two dedicated personnel and statistical analysis was conducted using SPSS 25.0 software. The scores of health-promoting lifestyle were described with mean ± standard deviation; t-test, ANOVA, and nonparametric tests were used for monofactor analysis of health-promoting lifestyle; and multiple stepwise regression analysis was performed for variables with statistical differences in monofactor analysis to explore the influencing factors of patients' health-promoting lifestyle, with the test level a_λ_ = 0.05. *P* < 0.05 was considered statistically significant.

## Results

### Health-promoting lifestyle levels of rural populace with high risk of cardiovascular and cerebrovascular diseases

The health-promoting lifestyle scores of rural populace with high risk of cardiovascular and cerebrovascular diseases ranged from 78 to 193, with a mean score of 125.55 ± 20.50. Among the six dimensions, nutrition had the highest score, followed by interpersonal relationships and self-actualization, and exercise had the lowest score. 11 cases (1.9%) were in the poor level of health-promoting lifestyle, 383 cases (65.5%) were in the average level, 164 cases (28%) were in the good level, and 27 cases (4.6%) were in the excellent level. The mean scores and ranking of each dimension are shown in Table 1.

**Table 1:** Health-promoting lifestyle scores of rural populace with high risk of cardiovascular and cerebrovascular diseases (n = 585)

Dimensions	Scoring range (points)	Mean score (points, $\overline{x}$ ± s)	Order
Total score of health-promoting lifestyle	52–208	125.55 ± 20.50	
Interpersonal support	9–36	23.39 ± 5.22	2
Nutrition	9–36	25.24 ± 2.69	1
Health responsibility	9–36	17.49 ± 5.09	5
Exercise	8–32	14.86 ± 4.66	6
Stress management	8–32	21.60 ± 4.42	4
Self-actualization	9–36	22.97 ± 4.82	3

### Family support scores of rural populace with high risk of cardiovascular and cerebrovascular diseases

The family support scale scores of rural populace with high risk of cardiovascular and cerebrovascular diseases ranged from 1 to 15, with mean score of 12.69 ± 2.08, among which, 526 cases (89.9%) had high family support and 59 cases (10.1%) had low family support.

### General psychological status scores of rural populace with high risk of cardiovascular and cerebrovascular diseases

The general health questionnaire scores of rural populace with high risk of cardiovascular and cerebrovascular diseases ranged from 0 to 7, with mean score of 0.26 ± 0.82 points; 15 cases (2%) were detected to indicate mental health status.

### International physical activity questionnaire scores of rural populace with high risk of cardiovascular and cerebrovascular diseases

Classified with reference to the physical activity level grouping criteria, the final results showed 341 cases (58.3%) with high physical activity, 170 cases (29.1%) with medium physical activity, and 74 cases (12.6%) with low physical activity.

### Monofactor analysis of health-promoting lifestyle affecting rural populace with high risk of cardiovascular and cerebrovascular diseases

Monofactor analysis showed that age, education level, marriage, monthly per capita household income, physical activity based on the IPAQ, family support function, carotid intima-media thickness, and blood pressure had an effect on health-promoting lifestyle scores, with statistically significant differences (*P* < 0.05). See Table 2.

**Table 2:** General information about rural populace with high risk of cardiovascular and cerebrovascular diseases on their health-promoting lifestyles ($\overline{x}$ ± s, points)

Factors	Number of cases	Score of health-promoting lifestyle	t/F/H value	*P* value	Factors	Number of cases	Score of health-promoting lifestyle	t/F/H value	*P* value
Gender			− 1.630	0.104	BMI			1.673	0.172
Male	257	123.99 ± 20.24			≤ 18.4	14	132.07 ± 23.86		
Female	328	126.77 ± 20.65			18.5–23.9	173	123.12 ± 20.13		
Age			2.350	0.019	24–27.9	288	126.83 ± 20.83		
≥ 65 years old	202	128.28 ± 20.91			≥ 28	110	125.19 ± 20.50		
< 65 years old	383	124.11 ± 20.16			Homocysteine			0.824	0.410
Education level			7.692	0.000	Normal	501	125.84 ± 20.29		
Illiterate	196	123.23 ± 21.72			High	84	123.85 ± 21.77		
Primary school	233	123.39 ± 18.11			Carotid artery ultrasound (carotid intima-media thickness)				
Middle school	118	130.01 ± 20.76			Normal	432	126.76 ± 20.74	2.411	0.016
High school and above	38	136.89 ± 21.77			Abnormal	153	122.13 ± 19.48		
Monthly per capita household income			33.46	0.000	Blood pressure			4.281	0.005
Below RMB 3,000	445	122.34 ± 18.04			Normal	219	122.66 ± 19.04		
RMB 3,001 ~ 5,000	114	135.96 ± 24.73			Stage 1 hypertension	290	128.40 ± 20.87		
Above RMB 5,001	26	134.85 ± 20.50			Stage 2 hypertension	65	124.18 ± 21.31		
Marital status			− 2.651	0.008	Stage 3 hypertension	11	116 ± 24.89		
Unmarried, Widowed, Divorced	85	120.13 ± 19.31			General health questionnaires			1.590	0.112
Married	500	126.47 ± 20.57			Positive	12	116.25 ± 20.67		
Physical activity as per IPAQ			18.493	0.000	Negative	573	125.75 ± 20.47		
Low	74	112.47 ± 16.18			Sleep			0.559	0.572
Medium	219	128.27 ± 20.23			Normal	56	123.25 ± 21.53		
High	457	127.03 ± 20.47			Not bad	140	124.93 ± 19.57		
Family support function			− 6.946	0.000	Very good	389	126.11 ± 20.69		
Low	59	108.64 ± 16.41							
High	526	127.45 ± 20.05							

### Multiple stepwise regression analysis of health-promoting lifestyle affecting rural populace with high risk of cardiovascular and cerebrovascular diseases

The multiple stepwise regression analysis result showed that the effects of education level, monthly per capita household income, physical activity based on the IPAQ, and family support function on health-promoting lifestyle were statistically significant (*P* < 0.05). See Tables 3 and 4.

**Table 3:** Assignment of independent variables for multiple stepwise regression

Independent variables	Assignment
Age	< 65 years old = 1; ≥ 65 years old = 2
Education level	Illiterate = 1; Primary school = 2; Middle school = 3; High school and above = 4
Monthly per capita household income	Below RMB 3,000 = 1; RMB 3,001–5000 = 2; above RMB 5,001 = 3
Physical activity as per IPAQ	Low = 1; Medium = 2; High = 3
Family support function	Low = 1; High = 2
Carotid intima-media thickness	Normal = 1; Abnormal = 2
Blood pressure	Normal = 1; Stage 1 hypertension = 2; Stage 2 hypertension = 3; Stage 3 hypertension = 4

**Table 4:** Multiple stepwise regression analysis of scores of health-promoting lifestyle affecting rural populace with high risk of cardiovascular and cerebrovascular diseases

Variables	Regression coefficient	Standard error	Standardized partial regression coefficient	t	p
Constant term	67.829	5.723		11.852	0.000
Family support function	17.030	2.603	0.250	6.543	0.000
Monthly per capita household income	8.632	1.450	0.228	5.952	0.000
Education level	2.946	0.877	0.128	3.358	0.001
Physical activity as per IPAQ	3.426	1.109	0.118	3.090	0.002

## Discussion

### Status of health-promoting lifestyle of rural populace with high risk of cardiovascular and cerebrovascular diseases

The results of this study revealed that the total score of health-promoting lifestyle of rural populace with high risk of cardiovascular disease was 125.55 ± 20.50, which was at an average level, and the mean scores of each dimension in descending order were nutrition, interpersonal support, self-actualization, stress management, health responsibility, and exercise.

1. The score for nutrition was the highest. With the national-level promotion of new socialist rural construction, the living standard of rural people has improved significantly, and they are paying increasing attention to their nutritional needs. It is consistent with the findings of Wu et al. who found that most older people in rural areas were more actively aware of their nutritional needs and had a certain level of knowledge and understanding [15]. Healthy diet and proper nutrition play an important role in the primary prevention of cardiovascular and cerebrovascular diseases [16, 17]. Studies have shown that poor dietary habits are an important risk factor for the occurrence of cardiovascular and cerebrovascular diseases [18, 19]. Therefore, it is vitally important for primary health care providers to enhance education on healthy diet for rural populace with high risk of cardiovascular and cerebrovascular diseases.
2. The score of exercise is the lowest, which is related to the fact that 7 of the 11 administrative villages where the respondents of this study are located include a large number of people who have resettled, and with the improvement in farmers' living conditions, many of them are no longer relying on high-intensity farm work to survive as they once did. This is consistent with the findings of Hong et al. [20] However, numerous studies have demonstrated that insufficient physical activity and prolonged periods of sitting with less activity can increase the risk of cardiovascular and cerebrovascular diseases [21]. Therefore, primary health care providers should pay attention to the daily exercise of rural populace with high risk of cardiovascular and cerebrovascular diseases, and guide them to adopt correct exercise methods, so as to improve the overall level of health-promoting lifestyle [22].

### Influence of demographic characteristics on health-promoting lifestyles of rural populace with high risk of cardiovascular and cerebrovascular diseases

The education level and per capita household income had a positive regression with the level of health-promoting lifestyle in this study.

1. The level of health-promoting lifestyle was higher when the education level of rural populace with high risk of cardiovascular and cerebrovascular diseases was higher, which is consistent with the findings of Rêgo et al. [23] The reason for this may be that people with a high level of education pay more attention to their own health, and their demand and understanding ability of health knowledge and implementation of a health-promoting lifestyle were stronger, while people with a low level of education had poorer awareness of health knowledge and understanding ability, and compliance with a health-promoting lifestyle was relatively insufficient. The participants in this study are farmers with generally low education level; 33.5% of them are illiterate. Therefore, primary health care providers should provide personalized and specialized health guidance according to the education level of rural populace with high risk of cardiovascular and cerebrovascular diseases, be more patient and supportive of people with low level of education, intervene using easily understandable language, and ensure that interventions and evaluations are repeated.
2. People with higher monthly per capita household income had a higher level of health-promoting lifestyle, which is consistent with the findings of Wan et al. [24] This may be attributed to the fact that high-risk populace with low per capita household income focus more on their livelihoods and neglect their health, while those with high per capita household income have more time to acquire health knowledge and have the financial ability to conduct regular physical examinations, and they watch TV, and read books and newspapers to enhance their awareness. Therefore, primary health care providers should pay more attention to the high-risk population with low per capita household income and strengthen health education of this population, to improve their health awareness and health-promoting lifestyles [25].

### Effect of physical activity on health-promoting lifestyle of rural populace with high risk of cardiovascular and cerebrovascular diseases

This study showed that physical activity and health-promoting lifestyle ability of rural populace with high risk of cardiovascular and cerebrovascular diseases were positively correlated, i.e., higher the level of physical activity, better the overall health-promoting lifestyle level. Physical activity itself is part of health promotion behavior, but different intensities of physical activity may produce different health effects [26]. The *Guidelines for Adult Physical Activity in China* states that low levels of physical activity have limited health promotion effects and a moderate increases in physical activity (time, frequency, and intensity) can produce greater health benefits [27]. Therefore, when providing health interventions to rural populace with high risk of cardiovascular and cerebrovascular diseases, caregivers should conduct activity level assessments and ensure individualized adjustments based on individual activity levels (time, frequency, and intensity) to achieve the best benefits of individual activities, and ultimately promote the enhancement of health-promoting lifestyle competencies.

### Effect of family support function on health-promoting lifestyle of rural populace with high risk of cardiovascular and cerebrovascular diseases

This study showed that family support function of rural populace with high risk of cardiovascular and cerebrovascular diseases was positively correlated with health-promoting lifestyle competence, i.e., the more family support they received the stronger their level of health-promoting lifestyle competence, which is consistent with the findings of Yu et al. [28, 29] Good family function plays an important role in many aspects [30, 31]. In terms of psychology, it can enhance individual self-esteem and feelings of being loved, reduce patients' anxiety and depression, and diminish the sense of shame. In terms of physiology, it can improve sleep, relieve pain, which can facilitate the formation of health promotion behavior, and delay the disease progression. Therefore, in clinical practice, caregivers should pay attention to family support functions, enhance good and effective communication among family members to make them understand, tolerate, help, and supervise each other, and thus improve their health-promoting lifestyle competencies.

## Conclusion

Although clinical treatments for rural and urban populations with cardiovascular and cerebrovascular diseases is the same, health interventions for different populations may vary depending on their lifestyle (i.e., diet, exercise), educational level, etc. which could be obtained from our questionnaire-based survey. The health-promoting lifestyle of rural populace with high risk of cardiovascular and cerebrovascular diseases needs to be improved. In the process of nursing interventions, the health-promoting lifestyle level of populace with high risk of cardiovascular and cerebrovascular diseases should be comprehensively evaluated, the family function and individual activity level should be improved, and health interventions for people with low education level and low per capita monthly household income should be strengthened to assist the populace with high risk of cardiovascular and cerebrovascular diseases to establish and maintain a good healthy lifestyle and prevent the occurrence of cardiovascular and cerebrovascular diseases. The study is focused on farmers with high risk of cardiovascular and cerebrovascular diseases in specific rural areas, hence the scope is somewhat limited, and the sample has some local characteristics, therefore, the study of influencing factors needs to be expanded further for in-depth exploration.

## Data Availability

All data generated or analysed during this study are included in this article. Further enquiries can be directed to the corresponding author.
